# Severe ARDS Complicating an Acute Intentional Cresol Poisoning

**DOI:** 10.1155/2019/6756352

**Published:** 2019-08-20

**Authors:** Djoudline Doughmi, Lamiae Bennis, Aicha Berrada, Ali Derkaoui, Abdelkrim Shimi, Mohammed Khatouf

**Affiliations:** Department of Anesthesiology and Intensive Care A1, Hassan II Academic Hospital, Fez, Morocco

## Abstract

Cresol is a phenol derivative used as a disinfectant that may cause gastrointestinal corrosive injury, central nervous system, cardiovascular disturbances, renal, and hepatic injury following intoxication. We present a case of a female patient who was admitted to the emergency department after ingesting an unknown amount of cresol; she was admitted with tachypnea, shortness of breath with low oxygen level in the blood. She did not develop hepatic or renal dysfunction. The gastrointestinal endoscopy was performed and showed esophagus and gastric erosins only. The patient was sedated and ventilated for 7 days. After receiving supportive intensive care, the patient recovered and was sent for psychiatric evaluation. Cresol intoxication can be fatal, and cause a respiratory failure with an acute respiratory distress syndrome (ARDS), hepatic, and renal injury. This shows the importance of intensive care in the management of cresol poisoning.

## 1. Introduction

Cresol, a derivative of phenol is popularly used as a disinfectant and antiseptic. It is a mixture of the three isomers of methylphenol. After absorption through the skin and mucous membranes, it is metabolized by conjugation and oxidation. Its main toxicity is due to denaturation and precipitation of cellular proteins and thus poisons all cells directly. It can be absorbed following inhalation, oral, or dermal exposure. Its poisoning can be fatal. Cresol is extremely corrosive and may cause cutaneous damage, and gastrointestinal corrosive injury. In addition, Cresol is able to cause system central nervous system and cardiovascular disturbances, respiratory distress, hepatotoxicity, and nephrotoxicity.

## 2. Case Report

A 26-year-old woman with no medical history was admitted to the emergency department after attempting suicide by ingesting an unknown amount of cresol.

On admission she was agitated, a Glasgow coma scale of 14 was noted. She had no complaints except for vomiting. Her vital signs were a heart rate of 110 beats/min, a blood pressure of 135/73 mmHg, a respiratory rate of 30 breaths/min, and a blood saturation of 85% under 6 L oxygen supplement/min. Physical examination showed no skin discoloration, and no dark urine. An orotracheal intubation was performed.

The electrocardiography did not show any signs of repolarisation. Chest X-ray revealed a diffuse bilateral alveolar and perihilar infiltrates, a feature consistent with pulmonary edema. Echocardiography was normal. Laboratory tests did not show any abnormalities in her liver enzymes (alanine transaminase, aspartate transaminase, gamma-glutamyl transpeptidase, alkaline phosphatase, and lactate dehydrogenase were normal), nor a kidney injury; her creatinine level was normal. She did not develop haemolysis or rhabdomyolysis. Urine or blood dosage of cresol were not performed. Furthermore, a gastrointestinal endoscopy was performed and showed esophagus and gastric erosins only. Arterial blood gas analysis showed a pH of 7.32, a pCO_2_ of 29 mmHg, a bicarbonate level of 18 mmol/l, and a pO_2_ of 51 mmHg, with pO_2_/FiO_2_ ratio of 51 mmHg. Acute respiratory distress syndrome (ARDS) was concluded. She was sedated using an infusion of Midazolam (0.1 mg/kg/h) and Fentanyl (2 *µ*g/kg/h), ventilated with a tidal volume of 6 ml/kg body weight, positive end-expiratory pressure (PEEP) of 8 mmHg, and respiratory rate of 18 breaths/min, according to the latest recommendations.

**Figure 1:**
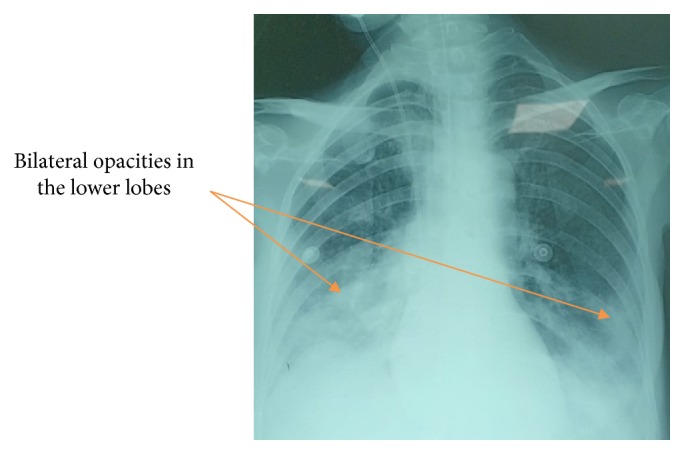
Chest X-ray on the 10^th^ day of admission showing bilateral opacities in the lower lobes (arrows).

On the 10^th^ day of her admission, she presented a septic shock secondary to nosocomial pneumonia to Acinetobacter Baumannii (Figure 1). She received tigecycline and colymicine.

**Figure 2:**
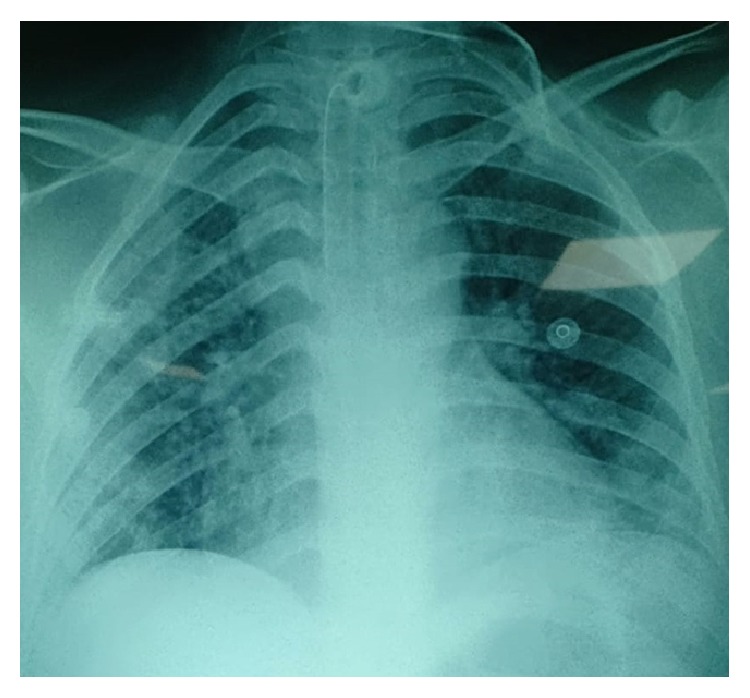
Chest X-ray on the 16^th^ day of admission showing a regression of the pneumonia.

After 7 days of sedation, and no respiratory improvement, we performed a tracheostomy. Our therapy included respiratory and motor kinesitherapy. Her chest X-ray was clean after 6 days of antibiotics (Figure 2), and her tracheostomy tube was successfully removed. The patient was sent for a psychiatric evaluation on the 18^th^ day of admission.

## 3. Discussion

Cresols, also known as “methylphenols”, are phenol derivatives, useful in a number of industrial applications and are effective antiseptics and disinfectants. Commercial cresol usually contains a mixture of *ortho*-methylphenol, *meta*-methylphenol, and *para*-methylphenol [1].

Cresol intoxication might be voluntary like in our case, or accidental. There are 38 reports of cresol intoxication with reported damage to multiple organs including the liver, lung, brain, and skin [2]. It can develop either after inhalation, cutaneous absorption, or oral ingestion. It is able to cause systemic effects such as neurological, cardiovascular, respiratory, gastrointestinal, hepatic, and renal injury.

Dermal exposure produces painless brown patches. When ingested, it causes esophagus and gastric erosins, nausea, vomiting, and diarrhea; the patient can develop severe gastrointestinal burns, but it is rare. Inhalation can cause irritation and pneumonia, sometimes acute respiratory distress syndrome can be seen, as we can see in our patient. The systemic manifestations include lethargy, coma, hypotension, tachycardia, acidosis, kidney, and hepatic injury.

The absorption, metabolism, and toxicity of cresol are quite similar to phenol's [3]. But its systemic effects may be milder due to a slower absorption [4]. Cresol is more corrosive than phenol and can cause dermal injury by protein denaturation and precipitation, leading to inflammation and necrosis of epidermal and dermal tissues.

The *p*-cresol is the most toxic of the three isomers (*o*-creso, *m*-cresol). After poisoning, cresol is rapidly detoxified by conjugation and excreted by kidneys or eliminated in the bile [5]. According to the results of animal experiments [6], for *p*-cresol, there is another metabolic pathway that leads via the oxidation of the methyl group to the formation of *p*-hydroxybenzoic acid. A reactive quinone-methide intermediate is formed, which can be bound to glutathione or macromolecules.

The diagnosis of cresol poisoning is based on a history of exposure, the odor of carbolic acid, clinical symptoms, painless brownish skin discoloration, and dark urine [7]. Blood and urine cresol levels and its metabolites can also be used to confirm the diagnosis [8], but it is not routinely performed.

The incidence of respiratory failure is low and the mechanism is still unknown. Cresol can cause bronchospasm and pulmonary edema in poisoned patients. Wu et al. [8] reported a case of a 44-year-old patient who was presented with a diffuse alveolar and perihilar infiltrates, a feature consistent with pulmonary edema after ingestion of 150 g of a 50% saponated cresol solution. The unusual location of pneumonia in bilateral apical lungs raised the possibility of cresol induced direct pulmonary toxicity. The follow-up for 8 months did not disclose any improvement of his chest X-ray, which indicated permanent lung injury. The clinical course of our patient was more severe than their case, our patient developed a severe acute respiratory distress syndrome requiring prolonged mechanical ventilation. However she did not develop kidney or hepatic injury; it can be explained by the fact that our patient was admitted agitated with vomiting; she could have developed aspiration pneumonia. Despite the small amount of cresol that she ingested, she was admitted only with respiratory failure.

Literature reported only one case of esophagus and stomach injury [9] with a gastrointestinal endoscopy after cresol poisoning revealing dark red corrosive injuries on the esophagus and stomach wall. Exploratory laparotomy was performed and showed edema and spotty bleeding on the serosa of the stomach. Total gastrectomy was required. A gastrointestinal endoscopy was performed on our patient, revealing esophagus and gastric erosins only.

The prevalence of renal dysfunction after cresol oral ingestion was 28.6% and the liver dysfunction was 62.5% [10]. The etiology of acute kidney injury could be the cresol direct nephrotoxicity, rhabdomyolysis, haemolysis, renal hypoperfusion following septic shock. The mechanism for the kidney direct toxicity may include tubular injury or capillary damage. Green [11] were able to determine the mechanism by renal biopsy in one fatal case; the renal failure was due to acute tubular necrosis. In our case, the patient did not show any sign of kidney injury despite the fact that she developed a septic shock. The fact that our patient did not develop a renal injury might be related to the small amount of cresol that she took.

Hepatic dysfunction is milder after cutaneous intoxication than oral ingestion. That was explained by Okammoto et al. [2], almost all the gastrointestinal absorbed cresol enter the liver via the portal vein, while cutaneous absorbed cresol may enter the systemic circulation without passing through the liver. The hepatic acinus (terminal acinus) can be divided into three zones in terms of metabolic actions. The centrilobular zone or zone III is more important for drug detoxification [12]. Cytochrome P450 catalyzes the oxidation of *para*-methylphenol to quinone methide; it forms a covalent that blinds to cellular protein and elicits hepatocellular toxicity [13].

Hayakawa [9] described a case about a 35-year-old patient who presented with a severe hepatic dysfunction after cresol poisoning; continuous hemodiafiltration was started and plasma exchange was needed. The patient had an uneventful recovery. Hashimoto et al. [14] described increases in aminotransferase levels after cresol intoxication but less severe than Hayakawa's [9].

Wu et al. noted one case of acute gouty attack after cresol ingestion [8]. It may be secondary to the hyperucemia resulting from his intravascular haemolysis and acute renal failure.

The management of cresol poisoning includes decontamination and supportive therapy; specific antidotes are not available. The excretion of cresol is mostly renal; enhanced elimination with hemodialysis or forced diuresis might decrease the toxicity of cresol. Charcoal hemoperfusion was also suggested and has shown its efficacy in one case of phenol poisoning [15].

The prognostic of cresol poisoning depends on the amount of cresol and its blood and urine levels. Our patient had an uneventful recovery because she took a small amount. A study conducted in 1969 on rats [16] showed that the median lethal dose (LD50) of *p*-cresol after oral ingestion was 207 mg/kg, after inhalation was 710 mg/kg, and after skin exposure was 750 mg/kg. Blood cresol levels of 71 and 120 *µ*g/ml have been associated with fatality, as mentioned by Green and Bruce et al. [11, 17].

## 4. Conclusion

Cresol intoxication can be fatal, causing systemic effects. In this case, the most prominent symptom was a respiratory failure with an acute respiratory distress syndrome. The patient did not show any sign of renal or hepatic injury, and she recovered with adequate therapy. This shows the important role of intensive care in the management of cresol poisoning.
